# Staged repair of a ruptured thoracoabdominal aortic aneurysm: a case report

**DOI:** 10.1186/s13019-024-02703-0

**Published:** 2024-04-15

**Authors:** Akitoshi Takazawa, Toshihisa Asakura, Hiroyuki Nakajima, Akihiro Yoshitake

**Affiliations:** Department of Cardiovascular Surgery, Saitama Medical University International Medical Center, 1298-1 Yamane, Hidaka City, Saitama Japan

**Keywords:** Ruptured aortic aneurysm, Endovascular, Staged repair, Graft replacement, Case report

## Abstract

**Background:**

A ruptured thoracoabdominal aortic aneurysm (rTAAA) represents a considerable challenge for surgeons. To date, endovascular procedures have not been able to completely replace open repair when debranching is required.

**Case presentation:**

A 73-year-old man was admitted to our hospital after complaining of left lateral abdominal pain. Enhanced computed tomography revealed a left retroperitoneal hematoma and a large, ruptured Crawford type IV TAAA. We first performed emergency resuscitative surgery to close the lacerated foramen. A graft replacement was performed 1 month after the initial surgery when the patient had stabilized. At 5 years postoperatively, neither occlusion nor anastomotic pseudoaneurysm was noted on computed tomography.

**Conclusions:**

We provide an update on the perioperative management of patients undergoing open rTAAA repair. This procedure can be considered to ensure complete repair of an rTAAA.

## Background

Open repair of thoracoabdominal aortic aneurysms (TAAA) is associated with a high mortality rate and is a major challenge for surgeons. In a report by Coselli et al. of 3309 open thoracoabdominal surgeries, postoperative mortality was 7.5% [1]. A national survey by the Japanese Association for Thoracic Surgery in 2014 reported that among 633 cases, 10.7% of hospital deaths were due to open thoracoabdominal surgery [2]. Aortic rupture represents the most lethal surgical challenge. Operative mortality ranges from 17 to 67% for ruptured TAAA (rTAAA), depending on whether the rupture is contained or free [3–8]. Almost all endovascular procedures have limitations. Therefore, open surgery remains the standard intervention for rTAAA. We report our experience with a staged repair of a ruptured type IV TAAA.

## Case presentation

**Fig. 1:**
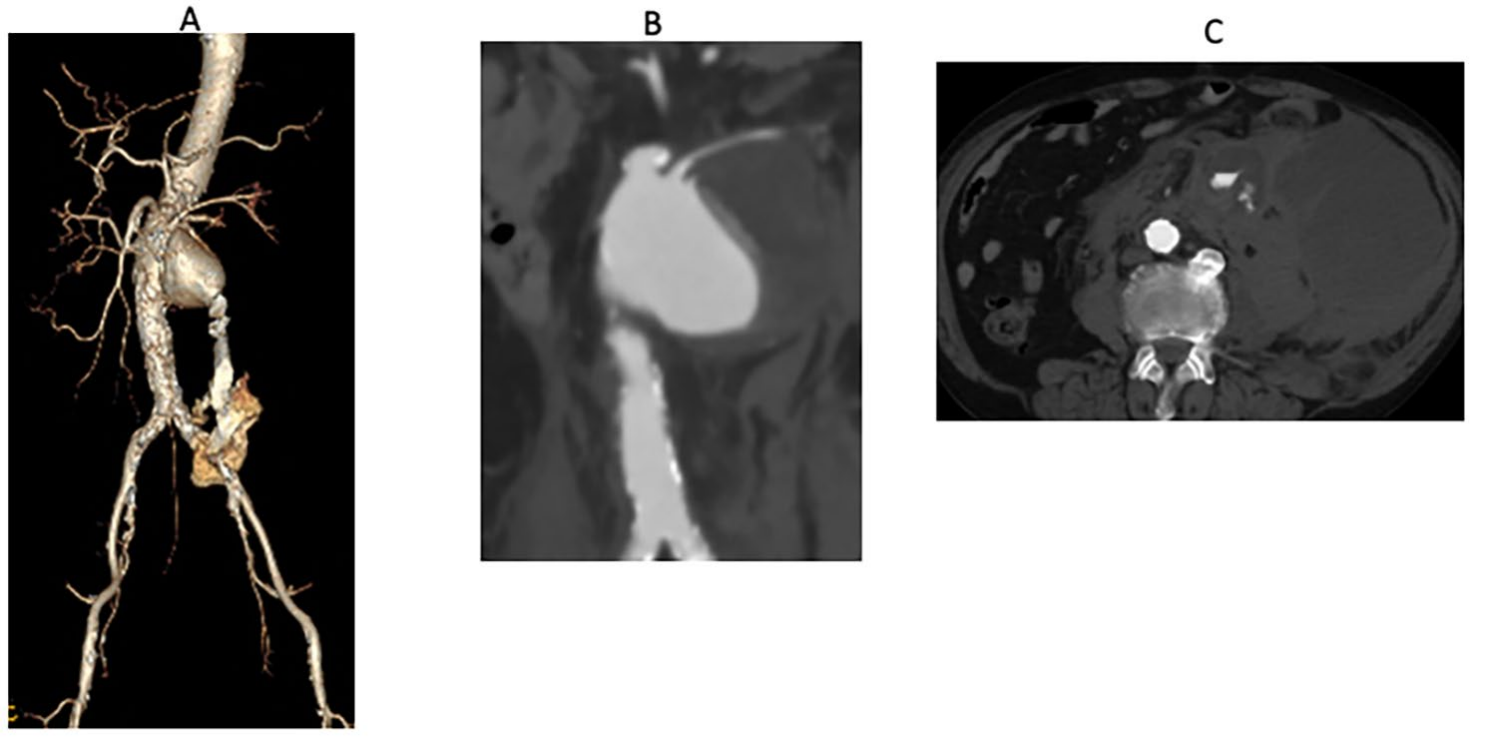
Preoperative CT. **A** and **B**: Preoperative computed tomography (CT) shows a ruptured thoracoabdominal aortic aneurysm (rTAAA) and the left renal artery arising from the thoracoabdominal aortic aneurysm (TAAA). **C**: The patient had a ruptured thoracoabdominal aortic aneurysm with a massive retroperitoneal hematoma

A 73-year-old man presented on foot and was admitted to our hospital with complaints of left lateral abdominal pain. Enhanced computed tomography (CT) revealed a left retroperitoneal hematoma and a large, ruptured Crawford type IV TAAA. The aneurysm was 80 mm in diameter. Furthermore, a tear in the left renal artery resulted from a ruptured aneurysm in the anterior wall (Fig. 1). The CT showed a heavily calcified visceral and infrarenal aorta, and the aneurysm was localized to the visceral segment, suggesting a mycotic penetrating atherosclerotic ulcer. The patient had not taken antibiotics. The preoperative evaluation revealed renal dysfunction (serum creatine, 1.21 mg/dL; estimated glomerular filtration rate, 46 mL/min) and no other comorbidities.

The patient’s level of consciousness suddenly deteriorated and his systolic blood pressure fell to 50 mmHg. Therefore, we performed endotracheal intubation and rushed the patient to the operating room. We chose open emergency surgery owing to uncontrollable hemorrhage, and we initially considered graft placement. A left thoracoabdominal incision is the standard approach; however, the patient’s hemodynamic status was exceedingly unstable. We expected this procedure, including arranging the operative position, to require more time than median laparotomy. Controlling the hemorrhage and stabilizing vital signs were of primary importance.

**Fig. 2:**
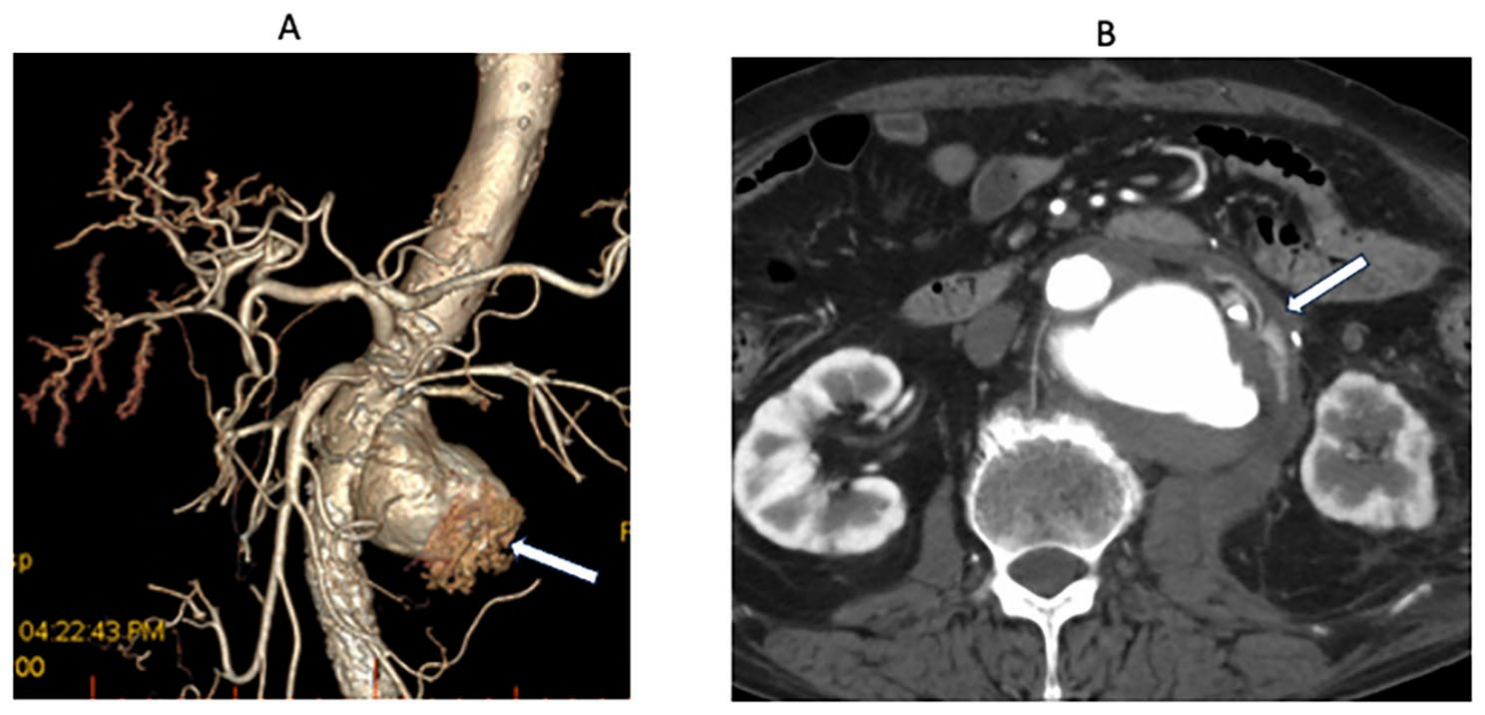
**A** and **B**: Computed tomography (CT) (day 12) after the initial operation shows the patch closure of the ruptured site (white arrow)

The patient was brought to a hybrid operating room and an occlusion balloon was inserted from the right femoral artery through the abdominal aorta under the diaphragmatic level. A median abdominal incision was made and intra-abdominal bleeding was observed. Even with balloon occlusion, bleeding was difficult to control and the surgical field was difficult to secure. Heparin was not administered. During dissection of the anterior wall of the aneurysm, bleeding was observed from the perforation, and without manual hemostasis, the patient’s blood pressure decreased to an undetectable level. The position of the central balloon occlusion was guided just above the aneurysm, and the rupture tear site was closed using 7 needles with 4 − 0 nonabsorbable monofilament, reinforced by pledget stitches (Bard polytetrafluoroethylene) to secure the operative field. This allowed the release of balloon occlusion, and the systolic blood pressure increased to 80 mmHg. Dissecting the proximal site of the aneurysm was impossible owing to the likelihood of hemorrhage. We considered open and patch closure of the aneurysm rupture site under balloon occlusion; however, considering the difficulty of maintaining an operative field and the risk of occluding the left renal artery, we elected to close the perforation site only and instead perform a 2-stage graft replacement after life salvage (Fig. 2). Intraoperative findings revealed no evidence of infectious aneurysm or pseudoaneurysm.

Because the aneurysm remained after the first surgery, the patient’s blood pressure was strictly controlled using medication. He remained hospitalized until the second surgery, ensuring urgent treatment if the aneurysm ruptured in the interim. Hemoglobin level and hematocrit were frequently measured (more than 3 days per week) and the TAAA was evaluated by CT once weekly. Stabilization of the patient’s condition required a full month, and the second surgery was then performed. He had no cardiac dysfunction (ejection fraction: 84%) or coronary artery disease according to the preoperative examination.

Under general anesthesia, the patient was placed in the left lateral position and a spiral skin incision was made. The aneurysm was approached by a left lateral thoracotomy through the seventh intercostal space, a diaphragm incision, and a pararectal approach. Severe adhesions were observed along the descending aorta from the celiac artery to the terminal abdominal aorta. A partial cardiopulmonary bypass was established by cannulation of the left femoral artery and vein. The descending aorta was cross-clamped under the diaphragm proximally and the left common iliac artery was controlled distally. The aneurysm was opened and the terminal aorta was clamped using an occlusion balloon. The thoracoabdominal aorta was replaced with the abdominal aorta under the celiac trunk immediately proximal to the aortic bifurcation. The superior mesenteric artery and the bilateral renal arteries were reconstructed with the interposition of a Carrel patch from small graft branches (24-mm Gelweave Coselli Thoracoabdominal Graft; Terumo Medical Corporation, Tokyo, Japan).

**Fig. 3:**
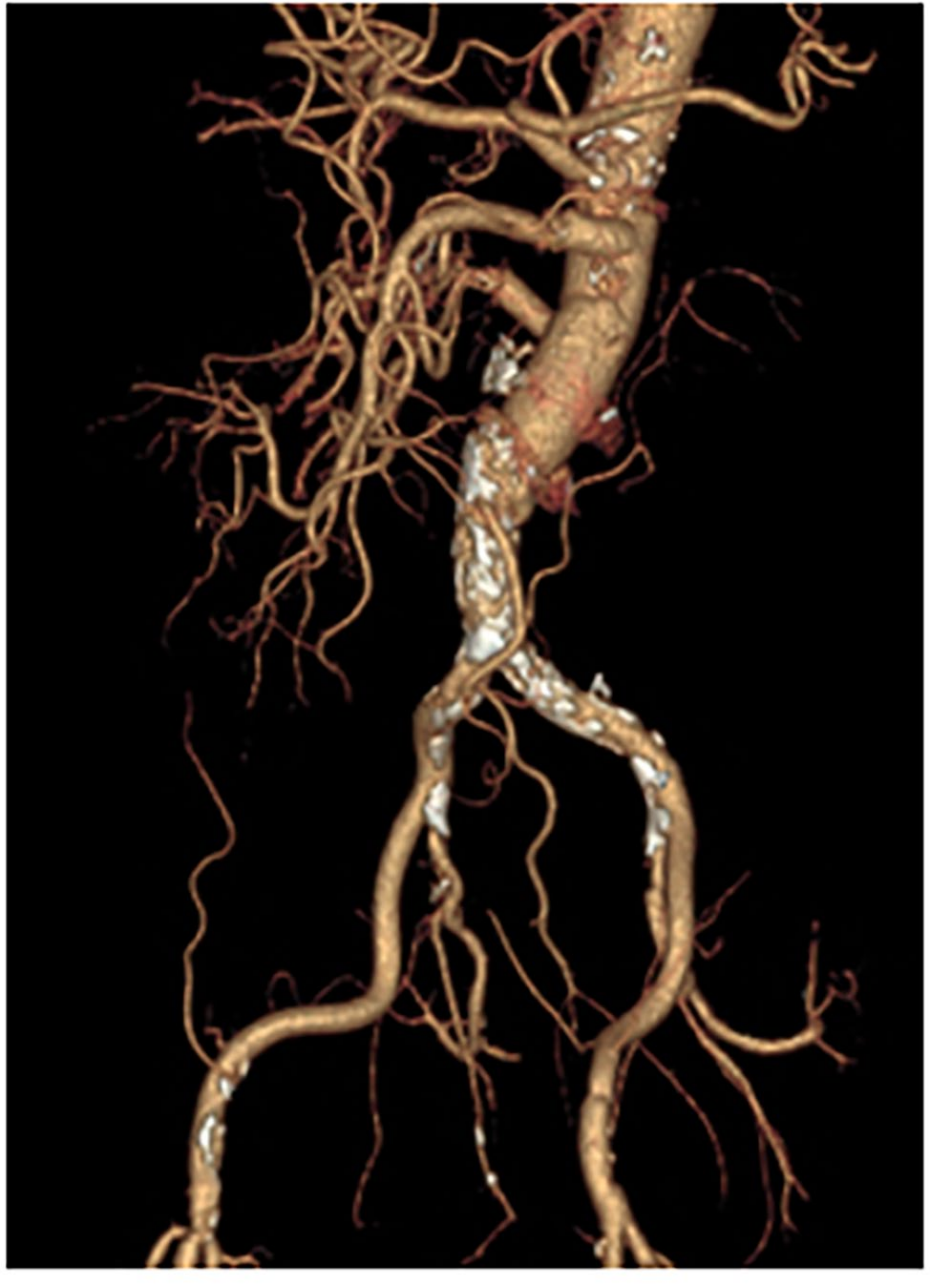
Computed tomography (CT) (5 years) after the second operation shows no pseudoaneurysm of the anastomosis. However, the graft to the left renal artery was occluded

Weaning from cardiopulmonary bypass and postoperative recovery were uneventful. The patient was removed from the intensive care unit on postoperative day 7. On postoperative day 12, CT revealed no anastomotic pseudoaneurysm, and the graft to the left renal artery was occluded (Fig. 3). The patient was discharged from our hospital on postoperative day 22. A recent follow-up CT scan, 5 years postoperatively, revealed no occlusion or anastomotic pseudoaneurysm.

## Discussion and conclusions

In a previous study, the postoperative mortality rate of rTAAA ranged from 40 to 70%, with no evidence of improvement in contemporary practice. More than half of these patients died within the first 24 h postoperatively [9]. The efficacy of rTAAA endovascular stent grafting has been documented in some case reports and small series reports [10–13]. These reports suggest that the incorporation of endovascular techniques may improve the otherwise excessive mortality rate that accompanies open surgical repair of rTAAA [9]. Hybrid therapy is also considered; however, it takes time for rupture to occur, and if an extravascular hematoma cannot be confirmed by CT scan, the results are poor, as shock is not indicated [14]. 

Published results from high-volume centers suggest that the reference standard method of treatment for all TAAAs is open surgery [15]. We agree that this is a particularly good option for type IV aneurysms in fit patients. Kondo et al. reported the successful replacement of a thoracoabdominal aortic graft after endovascular abdominal aneurysm repair for a ruptured infected aneurysm [16]. However, in our case, we were unable to opt for endovascular repair due to uncontrollable hemorrhage. In addition, an aortic cross-clamp was impossible because the left renal artery originated from the aneurysmal wall, and the aneurysm ruptured in the anterior wall, resulting in persistent retroperitoneal bleeding. Anatomically, graft replacement under cardiopulmonary bypass was necessary but not possible due to unstable hemodynamic status. Therefore, we performed the surgical intervention in two stages. First, emergency resuscitative surgery was performed with the primary goal of closing the lacerated foramen. This was followed by graft replacement 1 month later.

Unlike radical treatment, the disadvantages of this treatment were that the aneurysm remained, and that time had to pass for the patient to be stable enough for the second surgery, which represents a calculated risk. Our patient was kept hospitalized, and we were always prepared for a rapid response in case of re-rupture. Another disadvantage is that adhesions can form before secondary surgery. In the present case, severe adhesion was observed around the abdominal aorta on the peripheral side of the aneurysm, and dissection of the adhesion was difficult. Therefore, the peripheral side was clamped with a balloon and a peripheral anastomosis was performed.

Although our strategy in this case differs from that of general practice and guidelines, we believe that the use of a hybrid operating room was effective. A well-equipped hybrid operating room combines the capability of a traditional operating room with a wide array of devices and tools. This allows surgeons to perform various procedures and complex surgeries. A single-center retrospective review [17] of unstable ruptured abdominal aortic aneurysms comparing open conventional cross-clamping and endovascular balloon occlusion in a hybrid operating room reduced intraoperative mortality from 43 to 19%. However, a limitation of the hybrid operating room is the technical complexity requiring integration and coordination between different systems, such as surgical equipment, imaging modalities, and installation of specialized technology.

We report the successful management of an rTAAA in two stages, with an interval of 1 month. The initial open emergency procedure involved the closure of the ruptured site to stabilize hemodynamic status. The second procedure, a definitive open surgery that involved graft replacement, was performed 1 month later. This two-stage procedure can be considered as a method to ensure complete repair of an rTAAA with a lower risk of mortality.

## Data Availability

Data sharing is not applicable for this article, as no new data was created or analyzed in this case report.

## Abbreviations

CT: Computed tomography
rTAAA: Ruptured thoracoabdominal aortic aneurysm
